# HOLISTIC ASSESSMENT OF NURSING HOME RESIDENTS PREDICTS QUALITY OF LIFE AND PERCEPTION OF CARE

**DOI:** 10.1093/geroni/igad104.3391

**Published:** 2023-12-21

**Authors:** Emilee Ertle, Darby Simon, Benjamin Mast, Suzanne Meeks

**Affiliations:** University of Louisville, Louisville, Kentucky, United States; University of Louisville, Louisville, Kentucky, United States; University of Louisville, Louisville, Kentucky, United States; University of Louisville, Louisville, Kentucky, United States

## Abstract

The traditional medical model employs a problem-focused approach to assessment and care, but recent research emphasizes holistic assessment to facilitate person-centered care. The current study examines whether holistic assessment of problems and strengths can predict quality of life (QoL) and perception of care (PoC) of long-term nursing home residents. Sixty-six long-term nursing home residents were tested at time of admission, and at three and six month follow-up. Problem-focused measures were cognition, illness severity, depression, frailty, and anxiety. Strength-focused measures were hope, optimism, and social support. QoL at three months was correlated with anxiety (r=-.47, p=.001) and social support (r=.33, p=.047). At six months, QoL was correlated with anxiety (r=-.53, p<.001) and illness severity (r=-.30, p=.042). PoC at three months was not correlated with any problem-focused measures; it was correlated with optimism (r=-.38, p=.014), hope (r=.416, p=.008), and social support (r=.37, p=.036). PoC at six months was correlated with hope (r=.41, p=.009). Variables with significant correlations were selected for regression analysis; hope and optimism were combined into a single positive outlook variable. Anxiety was the only significant predictor in the regression model for QoL at three months. Anxiety and positive outlook were significant predictors of QoL at six months and PoC at three months. Positive outlook was the only significant predictor in the model for PoC at six months. Traditional problem-focused measures alone may provide insufficient information to predict well-being. Older adults’ strengths, particularly hope and optimism, may predict QoL and PoC in the months following nursing home admission.

